# Investigating Colorimetric Protein Array Assay Schemes for Detection of Recurrence of Bladder Cancer

**DOI:** 10.3390/bios8010010

**Published:** 2018-01-24

**Authors:** Selma Gogalic, Ursula Sauer, Sara Doppler, Claudia Preininger

**Affiliations:** Center for Health & Bioresources, AIT Austrian Institute of Technology, Konrad Lorenz Straße 24, Tulln 3430, Austria; selma.gogalic@gmx.at (S.G.); ursula.sauer@ait.ac.at (U.S.); sara.doppler@ait.ac.at (S.D.)

**Keywords:** bladder cancer, IL8, VEGF, DCN, colorimetric protein array, carbon nanoparticles

## Abstract

A colorimetric microarray for the multiplexed detection of recurrence of bladder cancer including protein markers interleukin-8 (IL8), decorin (DCN), and vascular endothelial growth factor (VEGF) was established to enable easy and cheap read-out by a simple office scanner paving the way for quick therapy monitoring at doctors’ offices. The chip is based on the principle of a sandwich immunoassay and was optimized prior to multiplexing using IL8 as a model marker. Six different colorimetric assay formats were evaluated using a detection antibody (dAB) labeled with (I) gold (Au) nanoparticles (NPs), (II) carbon NPs, (III) oxidized carbon NPs, and a biotinylated dAB in combination with (IV) neutravidin–carbon, (V) streptavidin (strp)–gold, and (VI) strp–horseradish peroxidase (HRP). Assay Format (III) worked best for NP-based detection and showed a low background while the enzymatic approach, using 3,3′,5,5′-tetramethylbenzidine (TMB) substrate, led to the most intense signals with good reproducibility. Both assay formats showed consistent spot morphology as well as detection limits lower than 15 ng/L IL8 and were thus applied for the multiplexed detection of IL8, DCN, and VEGF in synthetic urine. Colorimetric detection in urine (1:3) yields reaction signals and measurement ranges well comparable with detection in the assay buffer, as well as excellent data reproducibility as indicated by the coefficient of variation (CV 5–9%).

## 1. Introduction

Bladder cancer (BCa) is a serious malignancy of the urinary tract and prominent for its high rate of recurrence concerning 50% of all treated patients. To intervene recurrence of BCa, routine cytology and cystoscopy are done, representing the gold standard. Nevertheless, these methods are expensive, time-consuming, and invasive and lead to urinary infections in up to 16% of patients. Urine cytology has a high specificity (78–100%), although it lacks robust sensitivity (12.2–84.6%), especially for low and intermediate grade tumors, and is operator-dependent [1]. A number of urinary biomarkers (including fluorescence *in-situ* hybridization (FISH), ImmunoCyt, and nuclear matrix protein 22 (NMP22)) and investigational urine markers [2] also exist, but none of these markers has yet been shown to decrease the need for cystoscopy. Furthermore, biomarkers that aid the clinician in making treatment decisions are still an unmet need. Biomarkers identifying patients most likely to respond to chemotherapy for example would have enormous utility, not only reducing morbidity and improving quality of life but also significant cost savings to health providers. In previous work [3], we compiled a list of relevant bladder cancer protein markers based on extensive literature search (>1000 research articles) and prior defined inclusion and exclusion criteria. Microarray technology, a miniaturized high throughput analysis method, that can measure a high number of biomarkers in parallel was applied as a screening tool to narrow down the biomarker candidates and to evaluate the marker profiles in a clinical setting [4]. Ten potential protein biomarkers involved in the pathogenesis of the disease were identified and a respective biochip based on sandwich immunoassays and fluorescence detection was developed. Three (DCN, VEGF, and IL8) out of the 10 markers were significantly different (*p*-value < 0.05) in expression of protein levels concerning recurrent and non-recurrent BCa and were therefore considered highly suitable for detection of bladder cancer recurrence and therapy monitoring. As none of the three markers alone was specific and sensitive enough to allow for reliable detection, the three markers were combined in a multiplexed chip format, while the respective assays were transferred from a previously fluorescence-based to a colorimetric platform.

Using colorimetric detection can drastically reduce instrumentation costs and enable fast and easy read-outs by a commercial office scanner, digital camera, or even smart phone. This in turn promotes broader availability and deployment at doctors’ offices and more convenient therapy monitoring, since measurement is not invasive. It is also very user-friendly, as therapy monitoring, similar to glucose monitoring, can even be performed by the patient using a smartphone. Moreover, such colorimetric multiplex assays can be efficiently implemented in point-of-care (POC) settings.

In colorimetric assays, the analyte is determined with the aid of a color molecule, which is measured either as a colored solution or a colored precipitate depending on the technology platform. Using ELISA plates or microfluidic chips, the color is measured in solution in wells and lanes catalyzed by enzymes (e.g., horseradish peroxidase (HRP)) and optionally enhanced by gold nanoparticles through mechanisms, such as aggregation, controlled growth kinetics, metallization, and etching of metal nanoparticles (plasmonic ELISA) [5]. The color development relies on the biocatalytic reaction mediated modulation of local surface plasmon resonance (LSPR) properties. pELISA has enabled naked eye detection at very low concentrations of various clinically relevant biomarkers and pathogens [5]. Colored precipitates by contrast are typically detected on paper-based sensor arrays, with lateral flow devices or microarrays using enzymes, gold, or carbon nanoparticles (NPs). Enzymatic mimics, like Pd nanoparticles for detection of sarcosine in human urine samples, have also been introduced [6].

The prevalence of utilizing enzymatic labels, such as HRP and alkaline phosphatase (AP), is due to their high catalytic activity and selectivity based on specific enzyme–substrate binding. Furthermore, both the enzyme and the detection substrate are economical. Limitations of enzymatic colorimetric systems compared with detection using Au or carbon NPs are non-specific color precipitation and poor signal-to-noise ratios. Typical enzyme substrates are 3,3′,5,5′-tetramethylbenzidine (TMB) [7] or 3,3′-diaminobenzidine tetrahydrochloride (DAB) and 5-bromo-4-chloro-3′-indolyphosphate p-toluidine salt (BCIP) [8] for HRP and AP, respectively. Generally, the detection antibody is directly labeled with the enzyme [7] or via a biotin-streptavidin bridge.

Alternatively, gold and carbon NPs are used. Gold NPs are attractive labels due to their unique optical, electronic, chemical, physical, and catalytic properties [9,10], but costly in comparison with enzymes and carbon NPs. They can be produced in a wide size range between 2 and 200 nm and are easily functionalized with detection molecules forming highly stable conjugates. In a study by Jiang L. et al. [11], a colorimetric protein microarray for the serodiagnosis of TORCH infections (Toxoplasmosis, Rubella virus, Cytomegalovirus, and Herpes Simplex virus) was developed using colloidal gold particles in the size of 10.51 ± 0.24 nm coupled with the detection antibody via adsorption. The detection limit of IgM antibody was estimated to be 12.5 pg for the immunogold assay and 0.25 pg in case of the Cy3-labeled biotin-streptavidin approach [11].

Besides gold, carbon NPs of various sizes (20–200 nm) and shapes (colloidal, nano strings, nano rods, nanotubes) are used as detection labels [12,13]. Carbon NPs were firstly used in lateral flow immunoassays in the 1990s, giving proof of its suitability as a diagnostic tool in combination with image analysis [14]. Due to the high level of agreement between lateral flow devices and microarrays, carbon NPs were also introduced as detection labels in protein arrays, pioneered by Aart van Amerongen et al. [15]. For protein binding, carbon NPs were modified with neutravidin.

Although the use of Au, carbon, and enzymatic labels in colorimetric strip and on-chip assays has been reported in several publications, there is no systematic work to our knowledge that compares the performance of different detection labels using the same type of assay and the same analyte under the same conditions. We therefore aim to evaluate HRP as well as carbon and Au nanoparticles as detection labels in an on-chip sandwich immunoassay for IL8, DCN, and VEGF to significantly improve assay performance and reach sensitivities similar to respective fluorescence assays in spiked urine. 

## 2. Materials and Methods

### 2.1. Materials

Chip platform used was the proprietary ARChip Epoxy (EP 02799374; US 10/490543) and ArrayIt**^®^** SuperNitro Microarray. The capture antibodies (cAB) were spotted using the ArrayIt Nanoprint contact spotter with the SMP5 pin. As assay buffer LowCross-Buffer (100 050, lot#100A895p) from Candor (Wangen, Germany) was used. Streptavidin (strp)–gold from *Streptomyces avidinii* (40 nm, 07211-1 mL, lot#BCBP7056V), Tween 20, sodium deoxycholate, BSA (A7906-100G, lot#SLBM3734V), 16-mercaptohexadecanoic acid (448303-5G, lot#MKBJ8615V), *N*-(3-Dimethylaminopropyl)-*N*′-ethylcarbodiimide hydrochloride (EDC, 03449-5G, lot#BCBN0142V) and NaCl (S5886-500G, lot#SLBM5570V), 3,3′,5,5′-Tetramethylbenzidine (TMB2, T0565-100 mL, lot#SLBK8103V), sodium phosphate (dibasic, 30412) and silver enhancer kit (SE100-1KT) were derived from Sigma (St. Lous, MO, USA). Borate buffer (11455-90) was derived from EMS. Tris (M151-500G, lot#2243C149) was purchased from Amresco (Solon, OH, USA). EDTA (1.12029) and nitric acid (65%) were derived from Merck (Vienna, Austria). Streptavidin HRP (T20936, lot#1351296) was derived from life technologies (Carlsbad, CA, USA). S-002 (TMB1) and S-011 (O-dianisidine-based, ODI) were from Seramun (Heidesee, Germany). Human recombinant IL8 (574204) and anti-IL8 (H8A5, 511502, cAB, mouse), biotinylated anti-IL8 (mouse, E8N1, 511404) and unconjugated anti-IL8 (mouse, E8N1, 511402) were from Biolegend (San Diego, CA, USA). Neutravidin (#31000) was derived from Thermo Fisher Scientific (Waltham, MA, USA). Recombinant human VEGF 165 protein (293-VE), human VEGF antibody (MAB293), human VEGF165 biotinylated antibody (BAF293), recombinant human decorin protein (143-DE), human decorin antibody (MAB1432) and human decorin biotinylated antibody (BAM1431) were purchased from R&D Systems (Minneapolis, MN, USA).

### 2.2. Chip Fabrication

IL8, DCN, VEGF capture antibodies (cAB) were diluted in printing buffer (1 × PBS (pH 7.2)/0.01% sodium deoxycholate) to 0.4 g/L. The antibodies were arrayed on Supernitro slides (ArrayIt**^®^**, Sunnyvale, CA, USA) using the Arrayit Nanoprint contact spotter. The spot-to-spot distance was 500 µm. Each probe was spotted in 9 replicates in 12 identical arrays per slide at a relative humidity of 50%. To allow full immobilization of the probes the slides were kept at 4 °C for at least three days.

### 2.3. Preparation of Immunogold Probes

1 mL of 40 nm gold particles (40 nm, CG-40-100, lot#2457227_40) from Cytodiagnostics (Burlington, VT, Canada) was centrifuged at 4 °C for 15 min using 2500 g and the supernatant was discarded. 100 µL of 0.1 mM 16-MHA in water was added and incubated for 18 h at 22 °C. For EDC activation, the carboxylated gold nanoparticles were centrifuged for 15 min and resuspended in 98 µL water and vortexed. 2 µL 50 mM EDC in water were added and vortexed. The particles were incubated for 30 min at 22 °C. Then they were centrifuged for 15 min and resuspended in 100 µL 0.1 g/L unconjugated anti-IL8 in water. The incubation was carried out at 22 °C for 2 h. After a final centrifugation step the pellet was resuspended in 100 µL TBS (20 mM Tris, 150 mM NaCl, pH 8.0) + 1% BSA.

### 2.4. Oxidation of Carbon Nanoparticles

200 mg of carbon Special Black 100 powder (51 nm) from Orion (Frankfurt, Germany) was dispersed in distilled water for 30 min under sonication. The resulting suspension was then refluxed in 10 mL of nitric acid at 120 °C for 3 h to obtain oxidized carbon nanoparticles (oCNPs) [16]. After centrifugation the solid sample was washed two times with deionized water and then dried at 100 °C for 18 h. Oxidation was proved by elemental analysis (SEM TM3030 with EDX, Hitachi, Feldkirchen, Germany) and Fourier-transform infrared spectra (FTIR) based on carbon black doped potassium bromide (KBr) pellets using a Spectrum 100 FT-IR instrument from PerkinElmer (Waltham, MA, USA) [17]. 

### 2.5. Antibody Coupling to oCNPs

1% (*w*/*v*) suspension of oCNPs was prepared and sonicated for 30 s. Then 100 µL of 0.5 g/L unconjugated anti-IL8, anti DCN or anti VEGF was added dropwise to 150 µL of 5-fold dilution of oCNPs in 5 mM sodium borate coupling buffer (pH 8.8) (0.2% *w*/*v*) and allowed to react for 18 h at 4 °C under gentle stirring. 

### 2.6. Modification of Carbon NPs with Neutravidin

1% (*w*/*v*) suspension of carbon NPs (used as received) was prepared and sonicated for 5 min. Then the 1% suspension was diluted 5-fold in 5 mM borate buffer and again ultrasonicated for 5 min. Next, 0.35 mg neutravidin was added to 1 mL of diluted carbon NP suspension and incubated for 3 h at RT on an over-head shaker (Biorotator, Biospec Products) [18].

Both probes from Section 2.5 and Section 2.6 were then washed (15 min, 13,600 g) at least three times with washing buffer (5 mM borate buffer + 1% BSA + 0.002% NaN_3_). Finally, the supernatant was removed and the pellet resuspended in storage buffer (100 mM borate buffer + 1% BSA + 0.002% NaN_3_) at a final concentration of 0.2% and stored at 4 °C till usage. Before use in the on-chip assay, the carbon particles were sonicated for 10 s.

### 2.7. Chip Processing

Surface blocking was done for 45 min in 1× PBS (pH 7.2)/0.1% Tween 20 in order to remove any unbound probes and to deactivate reactive surface groups. Slides were washed twice in 1× PBS (pH 7.2) and dried using compressed air. Slides were mounted into a Hybridization Cassette 4 × 16 (ArrayIt Corporation, Sunnyvale, CA, USA). 12 arrays/wells (7 mm × 7 mm) per slide were used. Each array was incubated with 50 µL calibration standard for 2.5 h. Spiked samples were prepared by serial dilution of mixes of analytes in assay buffer or synthetic urine (certified drug free urine (88121-CDF(F)) UTAK Laboratories Inc., Santa Clara, CA, USA) (1:3) (1 part urine, 2 parts assay buffer). Then the slides were washed three times with 1× PBS (pH 7.2)/0.1% Tween 20.

#### 2.7.1. Detection Antibody Labeled with Carbon-and Gold NPs

Slides were incubated for 45 min with 50 µL oCNPs labeled dAB (1:5 diluted in assay buffer) and gold NPs labeled dAB (1:5 diluted in buffer: 5% BSA, 0.15 M NaCl, 0.01 M sodium phosphate (pH 7) and 0.05% Tween). Finally, the slides (treated with oCNPs) were washed twice with 1× PBS (pH 7.2)/0.1% Tween 20, twice in 1× PBS (pH 7.2) and once in distilled water. Slides incubated with gold-labeled dAB were in addition incubated with 0.05 M EDTA for 5 min before silver staining (10 min, Section 2.8). All incubation steps were carried out on the orbital shaker (Stovall, Greensboro, NC, USA) at maximum speed and at room temperature. After washing the slides were dried using compressed air and stored in the dark until scanning.

#### 2.7.2. Detection via Streptavidin/Neutravidin-Biotin Binding

Slides were treated with 50 µL biotinylated dAB and further incubated with either neutravidin–carbon (1:20 dilution in assay buffer), strp-HRP (1:100 in assay buffer) or strp–gold (1:5 in buffer: 5% BSA, 0.15 M NaCl, 0.01 M sodium phosphate dibasic (pH 7.0) and 0.05% Tween 20) for 45 min.

Slides incubated with strp–gold were washed with 1× PBS (pH 7.2)/0.1% Tween 20, 1× PBS (pH 7.2), distilled water and incubated with 0.05 M EDTA for 5 min before silver staining (10 min, Section 2.8). The slides incubated with strp-HRP were also washed as before and treated with substrate (TMB or ODI) for 15 min. At the end all slides were washed twice with 1× PBS (pH 7.2)/0.1% Tween 20, twice in 1× PBS (pH 7.2) and in distilled water. All incubation steps were carried out on the orbital shaker (Stovall) at maximum speed at room temperature. After washing the slides were dried using compressed air and stored in the dark until scanning.

### 2.8. Silver Staining

For the silver enhancement the excess solution was wiped off, 50 µL/well silver solution were added and incubated for 10 min. Finally, the slides were fixed with 2.5% sodium thiosulfate solution for 3 min and washed with dH_2_O. The slides were dried using compressed air.

### 2.9. Data Analysis

The colorimetric signal intensity was measured using an office scanner (Epson Perfection V330 Photo). Data were analyzed with the Genepix 6.0 software. The mean signal values were calculated from nine background corrected data points. Data points that were out of the mean signal values ± the standard deviation (SD) were excluded in an outlier test. Calibration curves were set up with OriginPro 8 using the logistic fit. The limit of detection (LOD) and dynamic range of the comparative IL8 assays were determined visually.

## 3. Results and Discussion

### 3.1. Colorimetric Assay Schemes

**Figure 1:**
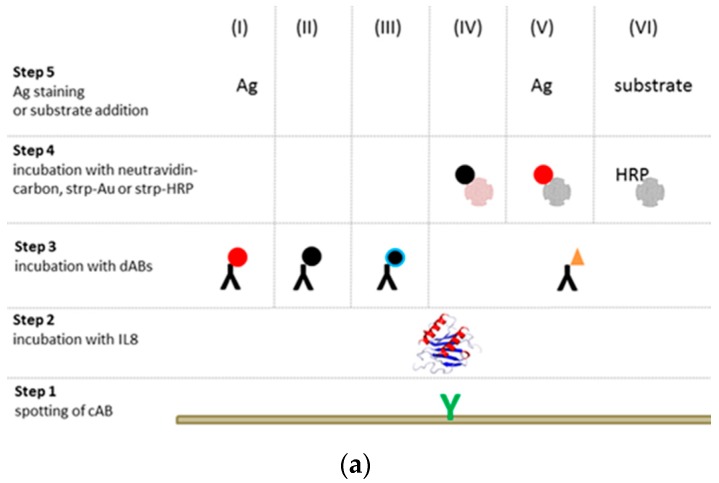

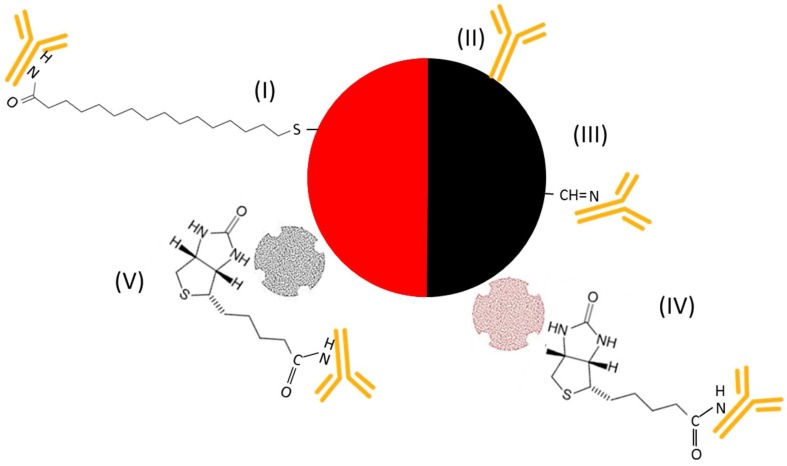
(**a**) Schematic presentation of six distinct assay formats for colorimetric detection: (I) cAB + IL8 + gold-labeled dAB, (II) cAB + IL8 + carbon-labeled dAB, (III) cAB + IL8 + oxidized carbon-labeled dAB, (IV) cAB + IL8 + biot. dAB + neutravidin–carbon, (V) cAB + IL8 + biot. dAB + strp–gold, and (VI) cAB + IL8 + biot. dAB + strp-HRP + enzyme substrate. Slides treated via gold nanoparticles were further stained by silver (Ag). (**b**) Illustration of the antibody (in orange) coupling process using gold (red) and carbon (black) nanoparticles.

Six colorimetric immunoassay schemes as described in Figure 1a were investigated in terms of sensitivity, reproducibility, signal intensity, and assay time to finally select the most sensitive one for the development of the multiplexed biomarker chip. Nitrocellulose slides were used as the chip platform. For cost reasons, we chose IL8 for evaluation of the proposed assay schemes. IL8 capture antibodies (cAB 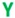) were spotted onto the chip to bind IL8 (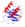) present in the sample solution (Step 2). For detection of the binding, the chip was, in a 3rd step, incubated with a detection antibody that was either labeled with NPs (I–III) or biotinylated (IV–VI 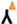). While incubation with an antibody labeled with (I) gold (AuNPs 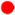), (II) carbon (CNPs 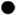), or (III) oxidized carbon nanoparticles (oCNPs 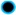) allows direct detection of the binding, the use of a biotinylated antibody requires additional incubation steps for signal detection. In Format (IV), Step 4, the biotinylated detection antibody reacts with neutravidin adsorbed on carbon nanoparticles (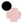); in Format (V), streptavidin is coupled with Au (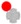); in Format (VI), streptavidin is linked to HRP (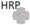). Additionally, Formats (I) and (V) are treated with silver (Ag), while Format (VI) is further processed with different enzyme substrates (Step 5).

AuNPs were modified using 16-mercaptohexadecanoic acid (MHA) (I), and the antibody was bound to the particle via EDC, a zero length crosslinker that covalently couples the carboxylic group of MHA to the amino groups of the antibody. CNPs were either used untreated for antibody immobilization (II) or oxidized (oCNPs) with nitric acid to introduce reactive quinone or conjugated ketone groups [17] for antibody binding (III). The neutravidin/streptavidin–biotin bridge was exploited in Formats (IV) and (V), so neutravidin and streptavidin, respectively, were adsorbed onto the NPs.

In total, we evaluated two colorimetric assay schemes using Au nanoparticles (Formats (I) and (V)), three using carbon nanoparticles (Formats (II)–(IV)), and one enzymatic approach (Format (VI)). A scheme of NPs modifications is shown in Figure 1b.

### 3.2. Assay Schemes with Nanoparticle-Labeled Detection Antibody

A comparative study was conducted using anti-IL8 labeled with Au and carbon NPs (Figure 1, (I)–(III)) to directly detect IL8 in the concentration range of 1 ng/L to 98.4 μg/L. 

Commercially available NPs were utilized to guarantee a high percentage of regularly shaped NPs preventing diffuse signals and generating well-confined and consistent spots [13]. Nitrocellulose with a 150 µm nitrosylated polysaccharide layer and a 0.20 μm porosity was applied. In order to work with colloidal stable [19] and sensitive AuNPs, 40-nm-sized particles were chosen. This is in good agreement with Nath et al. [20], who investigated the effect of particle size on the analytical sensitivity of an optical biosensor for streptavidin–biotin as a model receptor–ligand pair. The authors showed that the sensitivity of the sensor increases by a factor of three as size of Au NPs increases in the tested range of 13–47 nm. 

AuNPs have been coupled with anti-IL8 using MHA and carbodiimide [21]. MHA self-assembles on the Au particle forming a monolayer. Its carboxy group is subsequently activated with EDC to covalently bind IL8 through the amino groups present in the amino acids (arginine and lysine). Alternatively, the antibody might only be mixed with the AuNPs and adsorb on the particles via its thiol functions (cysteine). This was realized by Wang et al. [22], who coupled 40 nm Au particles to an anti-aflatoxin M1 antibody via adsorption.

**Figure 2:**
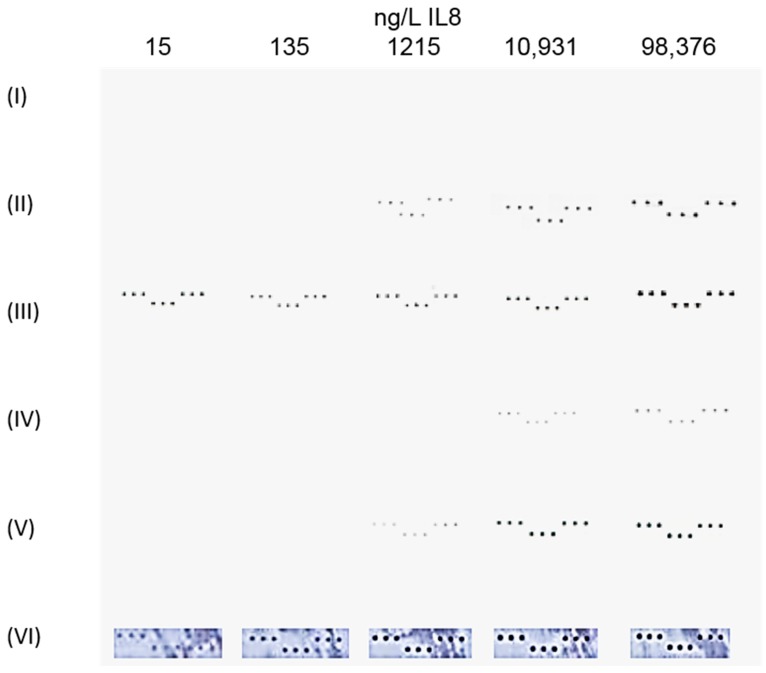
JPEG images of colorimetric subarrays after incubation with different labels based on Formats (I)–(VI). The corresponding IL8 concentrations are indicated in the columns as 15, 135, 1215, 10,931, and 98,376 ng/L.

In our study, the adsorption of antibodies on plain Au nanoparticles was not successful and the coupling antibody to Au via MHA led to mixed results with sometimes poor signals (Detection Scheme I) as can be seen from Figure 2. In the case of carbon NPs, brighter signals were obtained with oxidized carbon nanoparticles (oCNPs) (Detection Scheme III) than with carbon NPs that were implemented without further treatment (Detection Scheme II). This is most likely a result of higher functionality and hence more antibody bound to oCNPs compared to plain carbon. In fact, FTIR measurements revealed in the oxidized particles reactive groups, such as quinone or conjugated ketone [17], that are capable of binding with the amino acids of the detection antibody. Furthermore, elemental analysis data confirmed oxidation and showed increased oxygen values for oCNPs (9%) compared with CNPs (5%). However, in contrast to AuNPs both types of carbon NPs lead to visible spots, proving that Detection Scheme scheme III is more sensitive: the detection limit was 15 ng/L and improved by a factor of more than 10 compared to Detection Scheme II. Interestingly, Posthuma-Trumpie et al. [13] stated that adsorption is commonly used for carbon NP conjugation because it retains the specificity of proteins, important for antibody-antigen binding, and displays a fast and easy labeling procedure. This might be true for special types of carbon black but can by no way be generalized as there are plenty of different grades of carbon black commercially available made by different manufacturing processes in different sizes and surface areas. 

In this comparison, carbon NPs finally yield 10-fold higher assay sensitivity compared to AuNPs labels. The fact that carbon NPs used as labels can be more sensitive than gold NPs was already reported by Gordon et al. [23], who have undertaken a comprehensive survey of literature in PubMed. Thereby, sensitivities in the low pico-molar range were observed for carbon NPs, even by visual inspection. However, we have demonstrated that this is only the case when carbon NPs have been previously treated by nitric acid (oCNPs, Format (III)).

### 3.3. Assay Schemes with Enzymatic and Nanoparticles Labeled Avidin

#### 3.3.1. Evaluation of Enzyme Substrate

**Figure 3:**
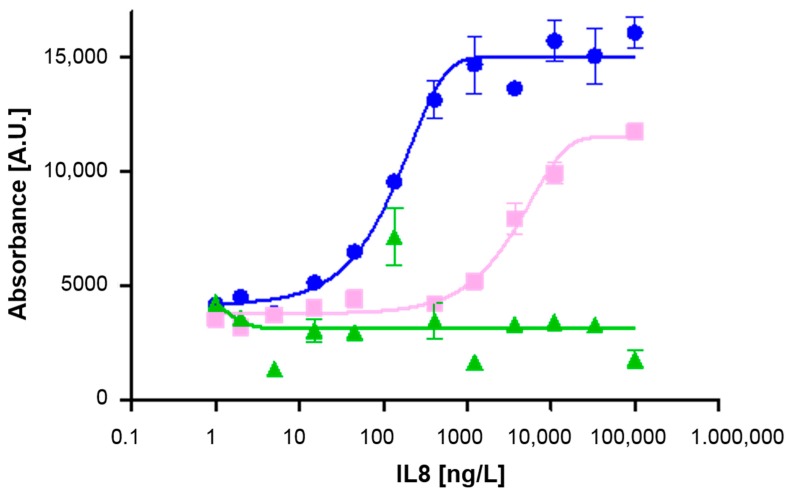
Mean readouts for marker IL8 using the enzymatic Assay Format (VI) in combination with different substrates: TMB1 (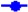), TMB2 (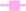), and ODI (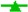). The blank (0 ng/L) is represented by 1 ng/L on the log scale.

Three enzyme substrates either based on TMB (from two different providers) or ODI were evaluated using Assay Format (VI). As shown in Figure 3, significant differences in assay performance were observed. As reported by the manufacturer ODI appears as a green precipitate after reaction with HRP and normally shows strong adherence on plastic surfaces. TMB precipitates as a dark blue color and is especially suitable for membrane surfaces, such as nitrocellulose.

Using the two different TMB substrates, the sensitivities, as defined by the LOD, were different by a factor of 15. The lowest detectable IL8 concentration was 15 ng/L with TMB1 and 405 ng/L with TMB2. This suggests that there are different qualities of enzyme substrate available in the market which all induce different reaction rates depending on the TMB and H_2_O_2_ concentration, pH and buffer system. Further influencing factors might be the solubility product of the precipitating agent with the TMB cation and the adhesion of the precipitate on the chip membrane. 

Data reproducibility as reflected by the small standard deviations was good with all tested substrates which points to a homogeneous staining pattern and very even distribution of precipitate within the spots. The enzyme substrate ODI was not suitable for this kind of assay, as no calibration curve was obtained. Our experience is actually in good agreement with Josephy et al. [24] who reported poor signal-to-noise ratio for colorimetric assays using ODI as the enzyme substrate arguing that one or more of the dianisidine products formed are unstable. In addition, the authors observed that ODI is short-lived and disappears within a few minutes [24]. This was not the case in our experiments.

In conclusion, TMB1 performed best among the tested enzyme substrates and was therefore used as substrate for HRP in all further investigations. 

#### 3.3.2. Detection Schemes Using the Streptavidin–Biotin Bridge

For detection of IL8, samples spiked with IL8 were incubated with the chip and the binding was detected with biotinylated antibodies and made visible in an additional step using Formats (IV)–(VI). To enhance the colorimetric signal of the gold NPs, the Au was stained with silver.

The enzymatic Format (VI) represents by far the most sensitive approach, with an at least 27-fold lower LOD compared to Assay Formats (IV) and (V). The good assay performance of the enzymatic approach (VI) is demonstrated in Figure 2, while spots visible with the naked eye are generated at much higher concentrations using Detection Schemes IV and V. 

Using Assay Format (V), bound streptavidin–Au was further enhanced with silver. Incubation time was 10 min, which led to 5-fold brighter signals compared to those without silver staining. As also reported by others, incubation time is critical: after 10 min, most silver was deposited; after 25 min, the silver enhancement reaction was in saturation [25]. Optimum incubation times reported are in the range of 10–15 min [26,27].

### 3.4. Comparison of the Six Protein Array Assay Schemes

**Figure 4:**
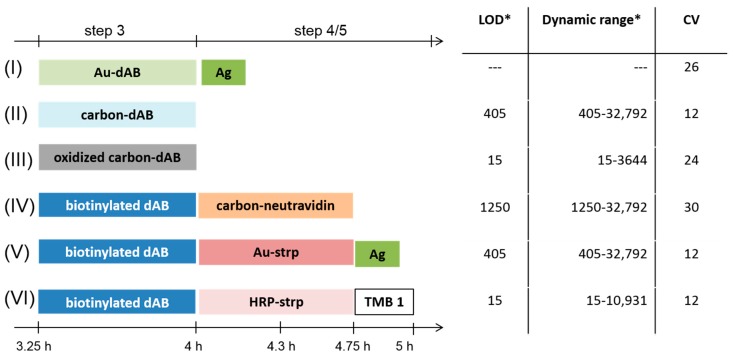
Schematic presentation of the detection of IL8 binding (Incubation Steps 3, 4, and 5) for tested Assay Formats (I)–(VI) including the limit of detection (LOD) (ng/L), dynamic range (ng/L), and coefficient of variation (%). * visually determined.

To identify the assay schemes that are both rapid and accurate, we compared the times required to fully complete the assay as well as the assay sensitivity, as summarized in Figure 2 and Figure 4. Assay Schemes (II) and (III) require three steps in total (spotting of the probes, incubation with the sample, and incubation with the labeled detection antibody) and represent the schemes with the shortest assay time (4 h). They are certainly the biomarker assays of choice in time-critical medical situations. Clearly, the assay time was not optimized, especially the incubation times of Steps 2–4 might be further reduced. In addition, Format (III) leads to the lowest LOD being 15 ng/L IL8, as low as Assay Format (VI). From Figure 2, it is obvious that those assay schemes were the only ones that could detect concentrations lower than 1215 ng/L IL8. Nevertheless, concentration-dependent black spots were obtained for all tested assay schemes except for Assay Scheme (I) (the Au nanoparticle-labeled detection antibody).

Results indicate that the choice of detection label strongly influences the assay sensitivity and measurement range of the immunoassay. This adds to the observations made with different types of nitrocellulose platforms (different modifications and different porosities) [28] or generally different types of surface materials [29] and enzyme substrates. Choosing the appropriate label allows the assay sensitivity to be tuned within a range that best fits the detection requirements, for example, to monitor the biomarker over several stages of the disease. In general, no clear trend towards the directly labeled detection antibody or the biotinylated detection antibody was observed. It is the label and its coupling efficiency, not the assay scheme, that governs assay sensitivity. 

Spot morphology is consistent for all assay schemes as indicated by the uniform size (90–120 µm) and appearance of the 3 × 3 replicate spots. The lowest LOD (15 ng/L IL8) was observed for Assay Format (III). Despite excellent sensitivity, a high BG was calculated for the enzymatic approach (Format (VI)), about 3.5-fold higher compared to the oCNPs assay (Format (III)). However, the background signal stays constant in Formats (III) and (VI). The high BG is likely due to non-specific precipitation of the colored product and has already been previously reported [30]. To overcome this problem, Liu et al. [30] established a washing strategy based on a ring-oven technique integrated on a paper-based immunodevice to measure carcinoembryonic antigen (CEA). By this method, the authors were able to effectively reduce non-specific binding and improve LOD 10-fold. However, this method requires also additional instrumentation and heating up to 80 °C, which might compromise antibody activity if not properly controlled at the detection zone.

Lönnberg and Carlsson [31] compared the detection ability of using colloidal carbon NPs (at the size of 51 nm) as an antibody label to those obtained with enzymatic labels. A nitrocellulose membrane-based immunochromatographic device and a flatbed scanner as a quantitative test system were used. The scanner detected 0.2–170 amol carbon black/mm^2^ with a coefficient of variation lower than 2% and an LOD of 0.02 amol/mm^2^. The detection ability of pure carbon was comparable to the method using horseradish peroxidase (HRP) together with TMB substrate yielding a colored signal of 5 amol HRP/microtiter well. These values are well comparable and about 2 orders of magnitude lower than those of, e.g. polystyrene particles [19]. In contrast to our study, the carbon NPs were adsorbed on the antibodies, because such a process is completed after only 30 min of incubation. In another article by van Dam et al. [32], the sensitivity of carbon NPs conjugates (prepared by adsorption) to detect Schistosomiasis was reported to be equal to that of the corresponding enzyme linked immunosorbent assay (ELISA).

Using the oCNPs (III) assay sensitivity was enhanced by 27 times compared to Scheme (V) using strp–AuNPs (V). This fits well with the results of Linares et al. [33] who demonstrated that carbon black had a remarkably low LOD of 0.01 g/L compared to 0.1 g/L and 1 g/L for silver-coated AuNPs and AuNPs, respectively.

### 3.5. Multiplexed Detection of IL8, VEGF and DCN in Assay Buffer and Synthetic Urine (1:3)

**Figure 5:**
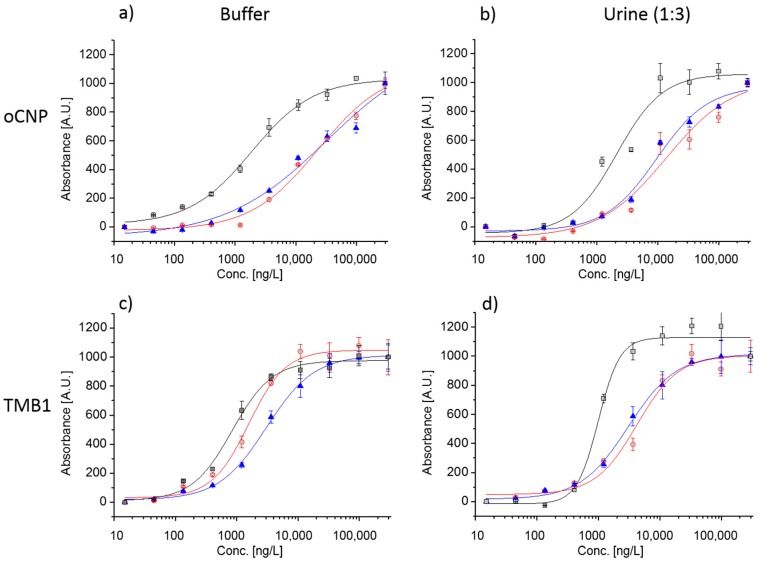
Normalized colorimetric signals in (**a**) assay buffer with labels oCNPs (Assay Scheme III); (**b**) in urine (1:3) with oCNPs (**c**) in assay buffer using HRP (Assay Scheme VI); (**d**) in urine (1:3) with HRP. Calibration curves for 0 to 295 µg/L: 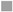 IL8, 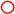 VEGF, and 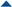 DCN; including standard deviations of three replicates. The blank signal (0 ng/L) is represented by 15 ng/L on the log scale.

Based on the outcome of Section 3.1, Section 3.2, Section 3.3 and Section 3.4 Assay Scheme (III) using oCNPs and Assay Scheme (VI) using HRP as a label in combination with TMB1 as substrate were further evaluated in a multiplexed chip format. Biomarkers IL8, VEGF, and DCN that were previously confirmed as important markers for detection of recurrence of bladder cancer [1] were spiked into assay buffer and synthetic urine (1:3) and incubated with the chip. The assay format for the three markers was the same as that described for IL8 in Section 2. The respective calibration curves are presented in Figure 5.

As is clear, the dynamic range of the assays is—despite the different signal levels—similar in buffer and in urine (1:3), and interferences by urine (1:3), such as low pH, urea, and other inhibitory components, are negligible. Measurement in pure urine, however, did indeed negatively affect assay sensitivity, and, depending on the protein biomarker, the limit of detection was enhanced by more than 10 times. Matrix effects from urine are reported to be differently pronounced for different assay markers. For example, Goodison et al. [34] evaluated a protein biomarker panel (IL8, MMP9, MMP10, ANG, APOE, SDC1, A1AT, PAI1, CA9, and VEGFA) in voided urine samples using a custom multiplex ELISA assay and divided the biomarkers into two sets, one to be measured with 4-fold diluted and one with 200-fold diluted urine samples. Data reproducibility as defined by the coefficient of variation (CV) was comparable for biomarker analysis in assay buffer and urine (1:3) and as low as 5 to 9%. Clearly, dilution of the urinary samples allows highly sensitive biomarker detection, but at the same time runs the risk to miss extremely low biomarker concentrations. Cut-off values reported in literature for IL8 and VEGF in urine were 25–35 ng/L, and 860 ng/L, respectively [3]. No cut-off value for DCN was found. Such low levels suggest that concentrators or signal enhancement kits shall be used to further improve assay sensitivity and meet the requirements for protein biomarker detection in therapy monitoring. Alternatively, several antibodies and antibody pairs might be tested in terms of sensitivity, specificity, physical properties, and recognition of native protein, and the best one selected for implementation in the multiplex chip [3]. 

## 4. Conclusions

Six protein array assay schemes and three different labels (Au, carbon, enzyme) were evaluated to identify the most sensitive colorimetric assay scheme to be exploited in an on-chip immunoassay for IL8, DCN, and VEGF, three protein markers that have been previously demonstrated to be important for the detection of bladder cancer recurrence. The highest signal intensity and assay sensitivity was observed for the enzymatic Assay Format (VI) with TMB1 as an enzyme substrate and Format (III) using oxidized carbon NPs (oCNPs). Herein, we could show that those schemes perform equally well in synthetic urine (1:3) and assay buffer in a multiplexed arrangement. With this colorimetric chip, a cost-effective, fast (4 h), simple, and efficient tool was established, which has high potential for use in doctors’ offices as a non-invasive tool in therapy monitoring.
